# Exploring the Impact of French Raw-Milk Cheeses on Oxidative Process Using *Caenorhabditis elegans* and Human Leukocyte Models

**DOI:** 10.3390/nu16121862

**Published:** 2024-06-13

**Authors:** Anna Diet, Christophe Poix, Muriel Bonnet, Christian Coelho, Isabelle Ripoche, Caroline Decombat, Julien Priam, Etienne Saunier, Pierre Chalard, Stéphanie Bornes, Florence Caldefie-Chezet, Laurent Rios

**Affiliations:** 1 Université Clermont Auvergne (UCA), Institut National de Recherche pour l’Agriculture, l’Alimentation et l’Environnement (INRAE), VetAgro Sup, Unité Mixte de Recherche Fromage (UMRF), F-15000 Aurillac, France; christophe.poix@vetagro-sup.fr (C.P.); muriel.bonnet@uca.fr (M.B.); christian.coelho@vetagro-sup.fr (C.C.); stephanie.bornes@uca.fr (S.B.); laurent.rios@vetagro-sup.fr (L.R.); 2 Université Clermont Auvergne (UCA), Centre National de la Recherche Scientifique (CNRS), Clermont Auvergne Institut National Polytechnique (INP), Institut de Chimie de Clermont-Ferrand (ICCF), F-63000 Clermont-Ferrand, France; isabelle.ripoche@sigma-clermont.fr (I.R.); pierre.chalard@sigma-clermont.fr (P.C.); 3 Université Clermont Auvergne, INRAE, Unité de Nutrition Humaine (UNH), Centre de Recherche en Nutrition Humaine Auvergne (CRNH-Auvergne), F-63000 Clermont-Ferrand, Franceflorence.caldefie-chezet@uca.fr (F.C.-C.); 4 Dômes Pharma, ZAC de Champ Lamet, 3 Rue Andrée Citröen, F-63284 Pont-du-Château, France; j.priam@domespharma.com (J.P.); e.saunier@domespharma.com (E.S.)

**Keywords:** raw-milk cheese, *Caenorhabditis elegans*, human leukocytes, oxidative stress, reactive oxygen species, antioxidant factors, stress tolerance

## Abstract

Fermented foods, including cheeses, have garnered increased interest in recent years for their potential health benefits. This study explores the biological properties of eight French raw-milk cheeses—goat cheese, Saint-Nectaire, Cantal, Bleu d’Auvergne, Roquefort, Comté, Brie de Meaux, and Epoisses—on oxidative processes using both in vivo (*Caenorhabditis elegans*) and in vitro (human leukocytes) models. A cheese fractionation protocol was adapted to study four fractions for each cheese: a freeze-dried fraction (FDC) corresponding to whole cheese, an apolar (ApE), and two polar extracts (W40 and W70). We showed that all cheese fractions significantly improved *Caenorhabditis elegans* (*C. elegans*) survival rates when exposed to oxidative conditions by up to five times compared to the control, regardless of the fractionation protocol and the cheese type. They were also all able to reduce the in vivo accumulation of reactive oxygen species (ROS) by up to 70% under oxidative conditions, thereby safeguarding *C. elegans* from oxidative damage. These beneficial effects were explained by a reduction in ROS production up to 50% in vitro in human leukocytes and overexpression of antioxidant factor-encoding genes (*daf-16*, *skn-1*, *ctl-2*, and *sod-3*) in *C. elegans*.

## 1. Introduction

In living systems, various metabolic processes and environmental stresses generate free radicals, particularly reactive oxygen species (ROS) [1,2]. In animals and humans, low and moderate amounts of ROS have beneficial effects on several physiological processes, including tissue repair processes, wound healing, and the killing of pathogens [3]. However, excessive levels of ROS, produced during oxidative metabolism, can damage the structure of biomolecules (especially DNA, proteins, lipids, and carbohydrates), modify their functions, and lead to cellular dysfunction or even cell death [4]. They are associated with accelerated aging [5] and inflammation [6] and are involved in the pathogenesis of several degenerative or chronic diseases such as atherosclerosis, osteoarthritis, cardiovascular or neurodegenerative diseases, and cancer [7,8].

The resultant imbalance between ROS production and their neutralization leads to a condition commonly referred to as oxidative stress [9]. To shield cells from ROS-mediated damage, organisms have several conserved endogenous defense mechanisms, such as the recruitment of antioxidant enzymes (superoxide dismutase, catalase, glutathione S-transferase, etc.). Therefore, to prevent oxidation in biological tissues, a variety of antioxidants can be employed. These antioxidants include both synthetic and natural substances able to neutralize and scavenge free radicals.

Intake of antioxidants through foods rich in these compounds or antioxidant supplements has the potential to protect the body from oxidative stress and associated damages. Over time, fermented foods have garnered significant attention in research concerning food sciences, nutrition, and health because, through the natural process of fermentation, they generate bioactive compounds of interest, including antioxidant compounds [10,11]. To increase food preservation and/or to improve organoleptic properties, fermentation has been used in a wide variety of food matrices, such as vegetables, cereals, soy, meat, and milk [12,13]. Such food processing increases the bioavailability of various constituents (phenolic compounds, bioactive peptides, antioxidant polysaccharides, etc.), which can exert positive biological effects after consumption. Bioactive peptides generated by fermentation from milk, whey proteins, or casein are studied for their beneficial biological activities such as antioxidant, anti-microbial, or anti-hypertensive activities [14,15]. Different studies have demonstrated that the antioxidant capacity of milk and dairy products is due to sulfur-containing amino acid cysteine compounds, vitamins (A and E), carotenoids, enzyme systems (catalase, glutathione peroxidase, superoxide dismutase), and polyphenolic metabolites [16]. In cheeses, these bioactive peptides are attributed to the chemical composition and the microbiota of the cheese [17,18,19,20], which are influenced by the wide variety of cheese-making processes, milk types, or ripening conditions [21,22,23,24,25,26]. Antioxidant activities have been reported on different types of cheese, such as white cheese [27], Parmigiano Reggiano cheese [28], Cheddar cheese [21,29], and Mexican goat cheese [30,31]. France is an important cheese production area, but few studies have been conducted to estimate the antioxidant activities of French raw-milk cheese using human cell cultures or animal models. One of our previous studies demonstrated that raw goat milk cheese (the entire cheese and cheese extracts) increased, at the same time, the capacity of the nematode *Caenorhabditis elegans* (*C. elegans*) to survive on an oxidative medium and decreased ROS production by human cells [32]. The similarities between *C. elegans* and human genetics, the conservation of the antioxidative response’s metabolic pathways, and the transparency of the body’s nematode make it a reliable experimental model to test the impact of such cheese fractions on the host [33].

Our study aimed to evaluate the potential antioxidant efficiency of eight raw-milk cheeses (Goat cheese, Saint-Nectaire, Cantal, Bleu d’Auvergne, Roquefort, Comté, Brie de Meaux, and Epoisses). Four fractions were prepared from each cheese: whole cheese and three extracts (one apolar and two polar extracts) to evaluate their impacts on (i) ROS production by human leukocytes and (ii) ROS accumulation, antioxidant response, and the survey of *Caenorhabditis elegans* placed on an oxidative medium.

## 2. Materials and Methods

### 2.1. Cheese Selection

Eight French cheeses made from raw milk were selected, considering characteristics such as the origin of the milk (animal), the type of paste, and the type of rind. The eight selected cheeses were representative of the main categories of French cheeses and had as varied characteristics as possible. To ensure the reliability and reproducibility of our study, we selected cheeses known for their high consistency in production and ample availability throughout the year. We chose the eight cheeses at their most popular and best-selling ripened stages (see Table 1).

### 2.2. Reagents and Solvents

Cyclohexane was purchased from Carlo Erba (Val de Reuil, France). 5-fluoro-2′-deoxyuridine (FUdR), amphotericin B, agarose, cholesterol, NaCl, MgSO_4_, CaCl_2_, NaHCO_3,_ Na_2_HPO_4_, NH_4_Cl, KH_2_PO_4_, EDTA, potassium phosphate buffer, gentamycin, phorbol 12-myristate 13-acetate (PMA), fetal bovine serum (FBS), glutamin, 3-(4,5-Dimethyl-2-thiazolyl)-2,5-diphenyl-2H-tetrazolium bromide (MTT), and NaOH were obtained from Sigma-Aldrich (Saint-Louis, MO, USA). Lysogeny Broth (LB, Miller’s Modification), peptone, and agar were purchased from Conda (Madrid, Spain). Yeast extract, Roswell Park Memorial Institute medium (RPMI-1640), and TRIzol Reagent were acquired from ThermoFisher Scientific (Waltham, MA, USA). 2′,7′-Dichlorodihydrofluorescein diacetate (H_2_DCF-DA) was purchased from Invitrogen (Carlsbad, CA, USA). Dihydrorhodamine 123 (DHR-123) was obtained from Cayman Chemical Company (Ann Arbor, MI, USA).

### 2.3. Obtention of Cheese Fractions

The cheese fractions were obtained from the eight cheeses as previously described [34]. Each cheese (with its rind) was cut into small slices. They were freeze-dried and crushed to a fine powder using a mortar and pestle. A part was conserved in waterproof containers as the first fraction: the freeze-dried cheese (FDC), and stored at −20 °C.

The rest of the FDC was mixed with distilled cyclohexane at a ratio of 1/10 (*w*/*w*) under mechanical stirring for 4 h. The solution was filtered with a Büchner funnel. The remaining filtrate was concentrated by evaporating the solvent under a vacuum to obtain a dry fraction. The solid resulting from the filtration was dissolved a second time in cyclohexane (ratio 1/10 (*w*/*v*)), mixed for 2 h, and filtered again. The solvent was evaporated from the solution under a vacuum to obtain a dry fraction, and the remaining solid was dissolved one last time with cyclohexane (ratio 1/10 (*w*/*v*)), mixed for 1 h, and filtered. The solvent of the solution obtained after filtration was evaporated to obtain a third dry fraction. The three dry fractions obtained from this process were pooled to obtain the cheese “apolar extract” (ApE).

The solid remaining from the cyclohexane extraction, which was collected through filtration, was extracted with HPLC-grade water (ratio 1/10 (*w*/*v*)). The mixture was mechanically agitated for 1 h at two different temperatures (40 °C or 70 °C) and centrifuged (15 min, 8000 rpm; Avanti J26S XPI, Beckman Coulter, Brea, CA, USA). The supernatant was recovered and evaporated under a vacuum. The solid obtained was dried under a vacuum. The cheese matrix was exhausted by repeating the same procedure three times under the same conditions. The resulting dry fractions were combined to form the final extract, ground using a mortar and pestle, and designated as either W40 (extraction at 40 °C) or W70 (extraction at 70 °C).

This methodology was applied to the 8 cheeses to obtain 8 freeze-dried cheeses (FDCs), 8 apolar extracts (ApEs), and 16 polar extracts (8 W40 and 8 W70). Each fraction was kept in waterproof containers at −20 °C.

### 2.4. In Vivo Study on Caenorhabditis elegans

#### 2.4.1. Growth of *Escherichia coli* and Heat-Killed Preparation

The *Escherichia coli* (*E. coli*) OP50 was provided by the Caenorhabditis Genetics Center (Minneapolis, MN, USA). The bacteria were grown overnight at 37 °C on a lysogeny broth medium as previously described [35]. After centrifugation (4000 rpm, 15 min; Rotofix 32A, Hettich Zentrifugen, Tuttlingen, Germany), the pellet was washed three times with M9 buffer (per L: 3 g of KH_2_PO_4_, 6 g of Na_2_HPO_4_, 5 g of NaCl, 1 mL of 1 M MgSO_4_), and the microbial suspension was adjusted to a final concentration of 100 mg/mL. The live *E. coli* OP50 was kept at 4 °C.

To obtain heat-killed (HK) *E. coli* OP50, the previous solution (*E. coli* OP50, 100 mg/mL) was held for 1 h in a water bath at 80 °C. The solution was kept at 4 °C until use.

#### 2.4.2. *Caenorhabditis elegans* Maintenance

The nematode *Caenorhabditis elegans* wild-type strain (N2) was acquired from the Caenorhabditis Genetics Center.

The growth and maintenance of nematodes were made as previsously described [36] on nematode growth medium (NGM) plates (per L: 3 g of NaCl; 2.5 g of peptone; 17 g of agar; 5 mg of cholesterol; 1 mM of CaCl_2_; 1 mM of MgSO_4_; 25 mL of 1 M potassium phosphate buffer at pH 6) supplemented with yeast extract (4 g/L) (NGMY). The NGMY plates were seeded with live *E. coli* OP50 as a source of food. The nematodes were kept at 20 °C during the maintenance [34,37,38].

#### 2.4.3. Synchronization of *Caenorhabditis elegans*

Synchronization of nematodes was performed at the beginning of each experiment, as previously described [34]. The eggs and the gravid worms were collected from NGMY plates, washed off using M9 buffer, and centrifuged (1500 rpm, 2 min; Rotofix 32A Hettich Zentrifugen, Tuttlingen, Germany). The worm pellet was resuspended in 5 mL of worm bleach (3.3 mL of M9 buffer; 700 µL of bleach with 12.5% sodium hypochlorite; 1 mL of sodium hydroxide 5 M) and vigorously shaken until adult worm bodies were disrupted. Forty milliliters of M9 buffer were added to block the effect of the worm bleach. The egg suspension was centrifuged (1500 rpm, 2 min) and washed twice with 20 mL of M9 buffer. The isolated eggs hatched in 20 mL of M9 buffer under slow agitation at 25 °C. After 24 h, the resulting L1 larvae were transferred onto NGMY plates, seeded with live *E. coli* OP50 as a food source, and maintained at 20 °C until the nematodes reached the L4 stage (young adult) [34,37,38].

#### 2.4.4. *Caenorhabditis elegans* Culture Conditions for Assays

An agar medium (per L: 3 g of NaCl and 6 g of agarose) was prepared and stored at 40 °C as previously described [32]. The medium was then split into aliquots, individually supplemented with 1% of cheese fraction (*w*/*v*). The aliquots were also supplemented with a nutritional source (50 µL of HK *E.coli* OP50 suspension at 100 mg/mL), FUdR (0.12 mM) to prevent the generation of progenies of *C. elegans*, and amphotericin B (1.6 µg/mL) to prevent any significant fungal development. The media for the control condition were the same, without cheese fraction supplementation. The aliquots were vortexed and poured into a 24-well plate (500 µL/well). The plate was immediately transferred onto ice to densify the agar and stored at 4 °C until use.

For experiments, approximately 25 synchronized N2 worms per well were incubated on the agar medium 24-well plate (NME plate), supplemented or not with cheese fraction, and kept at 20 °C for 5 days.

#### 2.4.5. Survival Rate of *Caenorhabditis elegans* on Oxidative Medium

The effect of the cheese fractions (FDC, ApE, W40, and W70) on the survival of *C. elegans* on an oxidative medium was determined as described in the previous study [32]. After 5 days of incubation on the NME plate, ≈15 worms per well were transferred onto an agar medium (per L: 3 g of NaCl and 6 g of agarose; “Free condition”) or with hydrogen peroxide (final concentration 3 mM; “H_2_O_2_ condition”) for 3.5 h.

The worm survival rate (Ꞇ) was determined using the following formula:

$$Ꞇ\;=\;\frac{{\left(\frac{n_{living\;worms\;at\;t=3.5\;h}}{n_{living\;worms\;at\;t=0\;h}}\right)}_{H_{2}O_{2\;condition}}}{\begin{array}{c} {\left(\frac{n_{living\;worms\;at\;t=3.5\;h}}{n_{living\;worms\;at\;t=0\;h}}\right)}_{Free\;condition} \end{array}},$$

The “Free condition” served as a control to ensure that the mortality observed in the oxidative medium was solely due to the addition of H_2_O_2_ and not anything else. The experiment was only considered valid when the survival rate in the “Free” medium was equal to 1 (100%). The nematodes were considered dead in the absence of a response to a mechanical stimulus. The effect of the fractions (FDC, ApE, W40, and W70) tested was evaluated in comparison with a control condition without cheese fraction supplementation. This assay was performed as 6 independent experiments conducted simultaneously with the control condition, with 3 wells per condition.

#### 2.4.6. Quantification of Intracellular ROS in *Caenorhabditis elegans* under Oxidative Conditions

The effect of cheese fractions (FDC, ApE, W40, and W70) on ROS accumulation in nematodes was measured using the probe H_2_DCF-DA as previously described [39] with slight modifications. The lipophilic, non-fluorescent H_2_DCF-DA is a sensitive cell-permeable redox probe. It crosses the cell membrane and is followed by deacetylation by cellular esterases in oxidant-sensitive dichlorofluorescein (DCFH). DCFH is oxidized later by ROS to form highly fluorescent dichlorofluorescein DCF, allowing us to quantify intracellular ROS levels in vivo.

After 5 days on the NME plate, the worms were washed in M9 buffer, transferred to a 24-well plate in M9 buffer with H_2_O_2_ (final concentration 1.5 mM), and subjected to continuous shaking at 100 rpm for 30 min. Afterward, H_2_DCF-DA was added to the wells (final concentration 50 µM) and incubated for 60 more minutes, protected from light, with 100 rpm continuous shaking. Following incubation, nematodes were immobilized using sodium azide (final concentration 0.5 M) and observed under a fluorescence microscope. Pictures of worms were made with a GFP filter (EVOS XL Core Imaging System, ThermoFisher, Hampton, VA, USA) and saved as PNG files.

On the 1140 photos obtained, the lack of contrast, the presence of undesirable elements (agar fragments, food residues, etc.), and the number of worms present per shot make automatic data extraction based on image analysis and pattern recognition difficult. We therefore carried out manual clipping on raw images for each worm identified. The resulting 1140 images were analyzed using Python scripts (with PIL—Python Image Library), which computed indicators such as average fluorescence, variance of fluorescence, or surface area in mm^2^ of each worm. The green light intensity in the pictures represents the accumulation of fluorescent probes in the worms. Rather than attempting to precisely quantify generated free radicals, our aim here is to compare the fluorescence of worms under various conditions.

For each condition, this assay was performed in at least 3 independent experiments with 10 worms per experiment. The fluorescence intensity of all the worms obtained (at least 30) gave the average fluorescence intensity per worm for each condition. This average was compared to the average of the control group.

#### 2.4.7. Quantitative Analyses of the *daf-16*, *skn-1*, *ctl-2*, and *sod-3* Gene Expression in *Caenorhabditis elegans*

L4-synchronized worms were cultivated for 5 days on NME plates supplemented or not with FDC (1%, *w*/*v*) and then exposed for one hour to M9 buffer with H_2_O_2_ (final concentration 0.75 mM) at slow agitation and room temperature. The mRNA expression levels of two transcription factors (*daf-16* and *skn-1*) and two antioxidant enzymes (*ctl-2* and *sod-3*) were measured quantitatively by real-time PCR after this exposure, following the protocol previously described [32]. Worms were collected and washed twice with M9 buffer. After centrifugation (1500 rpm, 2 min), the supernatant was removed, and 500 µL of TRIzol reagent was added to the worm pellet for RNA isolation. Worms were disrupted by using a Precellys (Bertin instruments, Montigny-le-Bretonneux, France) with glass beads (PowerBead Tubes Glass 0.1 mm, Mo Bio Laboratories, Carlsbad, CA, USA) and a succession of two sessions at 10,000 rpm for 30 s each. A break of 30 s on ice between the two sessions allowed the tubes to cool down. The tubes were centrifuged (14,000 rpm, 1 min) to remove the beads, and the supernatant was transferred to a new tube with 100 µL of chloroform. After 30 s of vortexing, the tubes were incubated at room temperature for 3 min. The samples were then centrifuged (12,000 rpm, 15 min, 4 °C), and the aqueous phase was transferred to another tube and treated again with chloroform under the same conditions. To precipitate the RNA, 250 µL of isopropanol was added to the aqueous phase, and the tubes were incubated for 4 min at room temperature before centrifugation (12,000 rpm, 10 min, 4 °C). The supernatant was discarded, and the pellet was washed with 1 mL of 70% ethanol. After centrifugation (14,000 rpm, 5 min, 4 °C), the pellet was dissolved in 20 µL of RNase-free water. The High-Capacity cDNA Archive kit was then used on 1 µg of RNA to perform a reverse transcription of the samples in accordance with the manufacturer’s instructions. For the real-time qPCR experiment, each sample tube contained 2.5 µL of cDNA, 6.25 µL of Rotor-Gene SYBR Green Mix, 1.25 µL of 10 M primers (Table 2), and 1.25 µL of RNase-free water. Each sample was run in triplicate. This study was performed using Rotor-Gene Q Series software 2.1.0 (Qiagen GmbH, Hilden, Germany). The gene Y45F10D.4 was used as the reference [40]. The 2^−ΔΔCt^ method was used to determine the relative changes in gene expression.

ΔΔCt = [(Ct_target_ − Ct_Y45F10D.4_) Sample] − [(Ct_target_ − Ct_Y45F10D.4_) Control]

### 2.5. In Vitro Analyses of Human Leukocytes

#### 2.5.1. Leukocyte Obtention

Blood was collected from healthy human volunteers (*n* = 83; Etablissement Français du Sang EFS, Clermont-Ferrand, France). All donors gave their written informed consent for the use of blood samples for research purposes under EFS contract n°22-106 (in accordance with the following articles L1222-1, L1222-8, L1243-4, and R1243-61 of the French Public Health Code). As previously described [41], each blood sample was hemolyzed with an ammonium chloride solution (155 µM NH_4_Cl; 12 µM NaHCO_3_; 0.01 µM EDTA), centrifuged (1000 rpm, 10 min, 4 °C), and the pellet of leukocytes was resuspended in Roswell Park Memorial Institute 1640 medium (RPMI-1640) supplemented with 10% (*v*/*v*) heat-inactivated fetal bovine serum (FBS), glutamin (2 mM), and gentamycin (50 µg/mL). Leukocytes were adjusted to 10^6^ cells/mL.

#### 2.5.2. Kinetics of ROS Production by Leukocytes

As previously described [42], leukocytes were stimulated with PMA to enhance ROS production in vitro. Only the fractions that were homogeneous in a culture medium or in DMSO were used to perform this experiment. Therefore, only the cheese extracts (ApE, W40, and W70) were tested in vitro on human leukocytes. ROS levels were measured using the DHR123 probe, a non-fluorescent compound integrated by cells and oxidized by ROS to form rhodamine123, a fluorescent molecule. Leucocytes were incubated with cheese fractions (W40, W70, or ApE; 0, 25, 50, 100, or 200 µg/mL), PMA (1 µM), and DHR123 (1 µM) in 96-well plates for 2 h at 37 °C. Fluorescence (excitation/emission: 485/538 nm) was recovered every 5 min for 2 h at 37 °C using the Spark^®^ reader (TECAN, Lyon, France). The results were presented as the percentage of ROS production in treated cells relative to the control cells (set at 100%).

#### 2.5.3. Leukocyte Viability MTT Assay

Leukocyte viability was measured with MTT, which is metabolized in blue formazan by the mitochondrial dehydrogenase of live cells. The following protocol [43] was used, with slight modifications: Two hundred thousand cells (200 µL) were incubated in 96-well plates with PMA (1 µM) and cheese fractions (ApE, W40, or W70) at 37 °C in a humidified 5% CO_2_ incubator. After incubation, plates were centrifuged (1 000 rpm, 6 min), and most of the media were removed. MTT reagent (0.5 mg/mL) was added to the wells, and the plates were incubated (4 h, 37 °C). The intracellular formazan product was dissolved in 150 µL of dimethyl sulfoxide (DMSO). After incubation (10 min, 37 °C), the absorbance of each well was measured at 540 nm using the Spark^®^ reader (TECAN, Lyon, France).

### 2.6. Statistical Analysis

Statistical significance was performed with GraphPad Prism version 8.2.1 for Windows (GraphPad Software, La Jolla, CA, USA). Groups were compared to the control using a two-tailed Student’s *t*-test. Differences were considered statistically significant at a *p*-value < 0.05. Differences between conditions were determined by using the Kruskal–Wallis test followed by an uncorrected Dunn’s test [44,45] and were considered statistically significant at a *p*-value < 0.05.

## 3. Results

### 3.1. Assessment of the Protective Effect of Cheese Fractions in C. elegans under Oxidative Conditions

**Figure 1:**
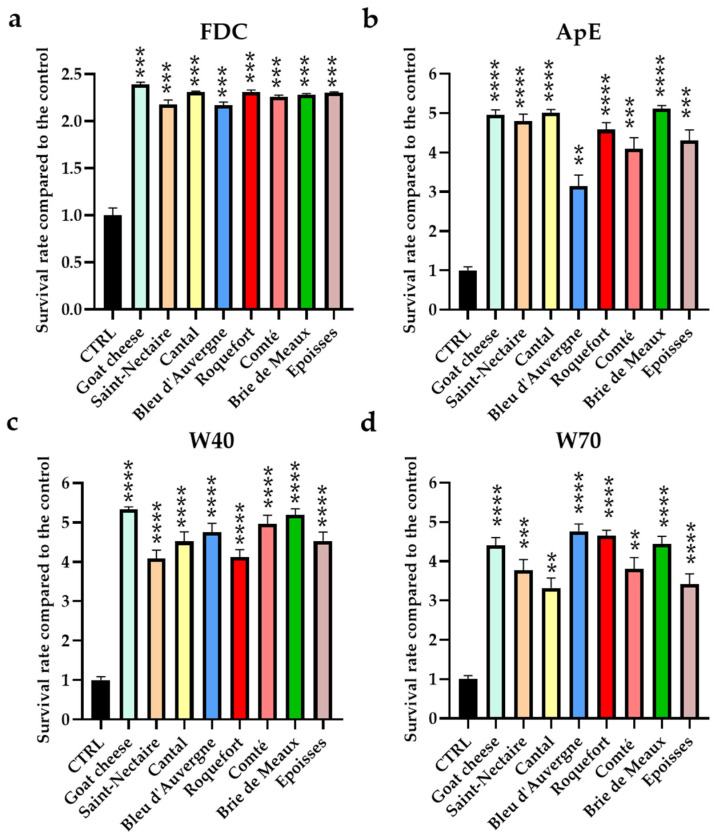
Survival rate of the *Caenorhabditis elegans* N2 strain on oxidative medium. Worms were incubated on a standard culture medium (CTRL) or supplemented with FDC (**a**), ApE (**b**), W40 (**c**), or W70 (**d**). After 5 days, worms were placed on agar medium with H_2_O_2_ for 3.5 h. The survival rate was measured for each sample and compared to the survival rate of the control (CTRL), fixed at 1. Results are presented as the mean ± SEM (*n* = 6); ** = *p* < 0.01; *** = *p* < 0.001; **** = *p* < 0.0001.

To investigate the effect of cheeses on the oxidative process, we conducted survival assays in worms supplemented with cheese fractions (FDC, ApE, W40, or W70) or without (CTRL) before exposure to an oxidative medium (Figure 1). The survival rate of *C. elegans* was measured after 3.5 h on an H_2_O_2_ agar medium and compared to the control survival rate (CTRL) previously set at the value of 1.

The results revealed that all cheese fractions improved the survival of *C. elegans* when exposed to oxidative conditions. Compared to the control, supplementation with each FDC (Figure 1a) enhanced the survival rate of *C. elegans* from 2.17 times (Bleu d’Auvergne; *p* < 0.001) to 2.35 times (Goat cheese; *p* < 0.001). The supplementation with ApE (Figure 1b) improved the survival rate from 3.14 times (Bleu d’Auvergne; *p* < 0.001) to 5.11 times (Brie de Meaux; *p* < 0.001) compared to the control. The same observation was made for each polar extract (W40 and W70; Figure 1c,d), which increased survival rates from 4.08 (Saint-Nectaire; *p* < 0.0001) to 5.33 (Goat cheese; *p* < 0.0001) for W40 and from 3.32 (Cantal; *p* < 0.01) to 4.77 (Bleu d’Auvergne; *p* < 0.0001) times the control for W70.

### 3.2. Effect of Cheeses on ROS Accumulation in C. elegans on Oxidative Condition

**Figure 2:**
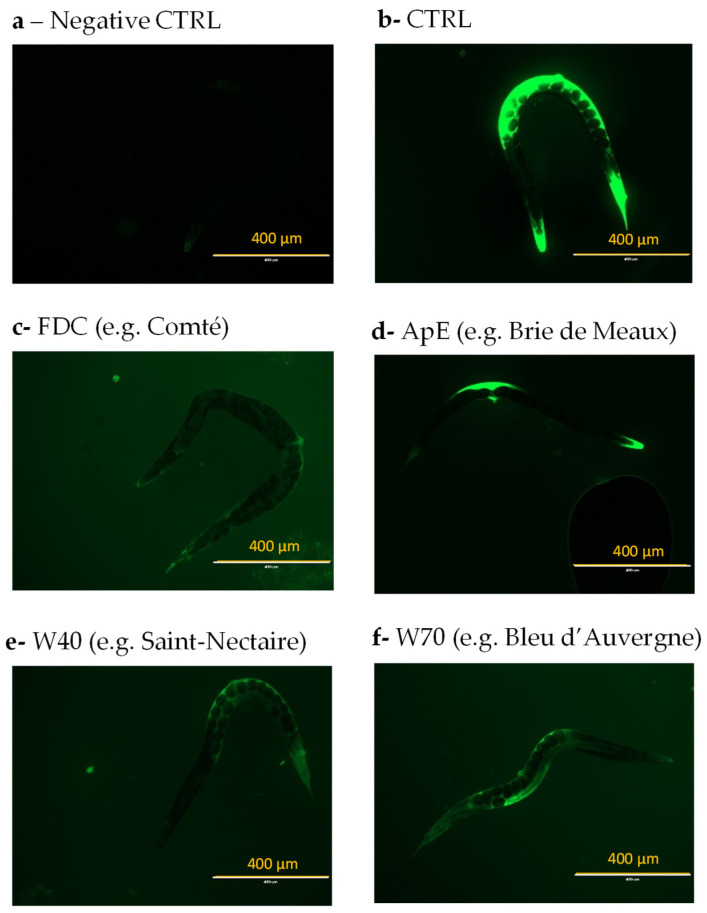
Representative GFP fluorescence microscopy images of ROS accumulation in vivo in *Caenorhabditis elegans* from different conditions. A wild-type N2 strain was cultivated for five days either on a standard culture medium (**a**,**b**) or supplemented with FDC (**c**), ApE (**d**), W40 (**e**), or W70 (**f**). After that, worms were placed on M9 buffer (**a**) or under oxidative conditions (**b**–**f**) and treated with H_2_DCF-DA to assess intracellular ROS levels in vivo. Images were acquired at 100× magnification.

We evaluated the effects of cheese fractions (FDC, ApE, W40, and W70) on ROS accumulation in *C. elegans* exposed to an oxidative environment (Supplementary Figures S1–S8). As expected, the worms cultivated on a medium without H_2_O_2_ (negative CTRL; Figure 2a) had less fluorescence intensity compared with the worms in the H_2_O_2_ environment (CTRL; Figure 2b). These results demonstrated that exposure to an oxidative environment induced the accumulation of ROS within the worm in vivo. In contrast, prior supplementation with cheese fractions (FDC, ApE, W40, and W70; Figure 2c–f) resulted in a reduced fluorescence intensity compared to the control.

**Figure 3:**
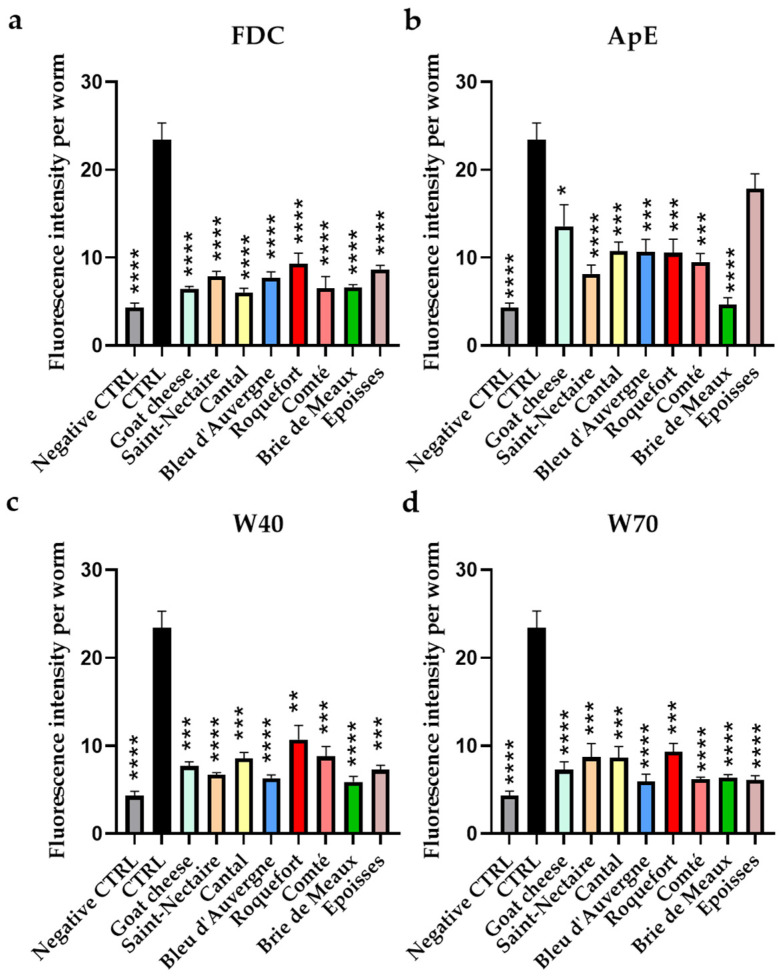
Impact of cheeses upon intracellular reactive oxygen species (ROS) accumulation in the N2 *Caenorhabditis elegans* strain. Worms were cultivated for 5 days on medium supplemented with FDC (**a**), ApE (**b**), W40 (**c**), or W70 (**d**). All conditions except the negative control (“Negative CTRL”) were then placed in the oxidative condition. All were treated with H_2_DCF-DA to assess intracellular ROS levels in vivo. Results are presented as the mean fluorescence intensity per worm ± SEM (*n* = 30). Samples are compared to the control in the oxidative condition (“CTRL”); * = *p* < 0.05; ** = *p* < 0.01; *** = *p* < 0.001; **** = *p* < 0.0001.

In comparison with the control worms subjected to H_2_O_2_ oxidative stress (without cheese fraction supplementation), we observed a reduction in ROS accumulation with the FDC (Figure 3a) from 60% for Roquefort (*p* < 0.001) and up to 72% for the four FDCs: Goat cheese (*p* < 0.0001), Cantal (*p* < 0.0001), Comté (*p* < 0.0001), and Brie de Meaux (*p* < 0.0001).

Apolar extract (ApE; Figure 3b) significantly reduced ROS levels by a minimum of 42% for Goat cheese (*p* < 0.05) and a maximum of 80% for Brie de Meaux (*p* < 0.0001). W40 polar extract (Figure 3c) contributed to a reduction in ROS accumulation ranging from 54% for Roquefort (*p* < 0.01) to up to 70% for the three FDCs: Brie de Meaux (*p* < 0.0001), Bleu d’Auvergne (*p* < 0.0001), and Saint-Nectaire (*p* < 0.0001). W70 polar extract (Figure 3d) reduced ROS amounts between 60% for Roquefort (*p* < 0.001) and 75% for Bleu d’Auvergne (*p* < 0.0001) compared with the control.

### 3.3. Impact of Cheese Extracts on ROS Production in Human Leukocytes

**Figure 4:**
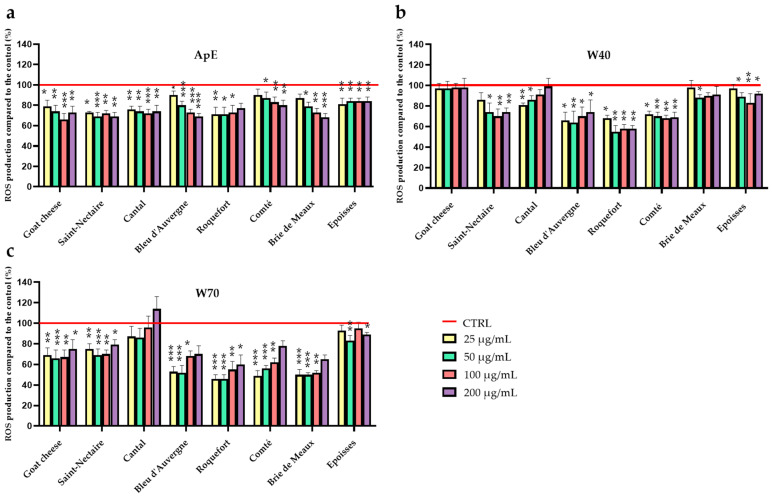
Effect of ApE (**a**), W40 (**b**), and W70 (**c**) upon reactive oxygen species (ROS) production on human leukocytes. Cells were incubated with fractions (25, 50, 100, or 200 µg/mL) and stimulated for 2 h to enhance ROS production. ROS production in the stimulated control was set to 100%. Results are presented as the percentage of ROS production in treated cells relative to the control cells (mean ± SEM; *n* = 6); * = *p* < 0.05; ** = *p* < 0.01; *** = *p* < 0.001.

To investigate the impact of the freeze-dried cheese extracts (ApE, W40, and W70) upon ROS production in cells, we performed an in vitro quantification experiment using PMA-stimulated human leukocytes to induce ROS production. Concurrently, cell viability was assessed using an MTT assay. The impact of the three extracts (ApE, W40, and W70) from each of the eight cheeses (24 extracts) on ROS production was compared with ROS production of the PMA-stimulated control (CTRL) cells set at 100% (Figure 4).

Twenty-two of the extracts tested showed a significant reduction in ROS production. Apolar extract (ApE; Figure 4a) of cheeses decreased ROS production from 16% for Epoisses (50 µg/mL; *p* < 0.05) to up to 31% for the five ApEs of Goat cheese (100 µg/mL; *p* < 0.001), Saint Nectaire (50 µg/mL; *p* < 0.001), Bleu d’Auvergne (200 µg/mL; *p* < 0.001), Roquefort (50 µg/mL; *p* < 0.05) and Brie de Meaux (200 µg/mL; *p* < 0.001), compared to the control. W40 polar extracts (Figure 4b) were able to mitigate ROS formation from 12% for Brie de Meaux (50 µg/mL; *p* < 0.05) to up to 45% for Roquefort (50 µg/mL; *p* < 0.01) relative to the control. W70 (Figure 4c) minimized ROS synthesis from 17% for Epoisses (50 µg/mL; *p* < 0.01) to up to 50% for the two W70’ extracts of Roquefort (50 µg/mL; *p* < 0.001) and Brie de Meaux (50 µg/mL; *p* < 0.001) in comparison with the control.

### 3.4. Effect of Cheese Supplementation on ROS Elimination in C. elegans through Antioxidant Pathway Activation

**Figure 5:**
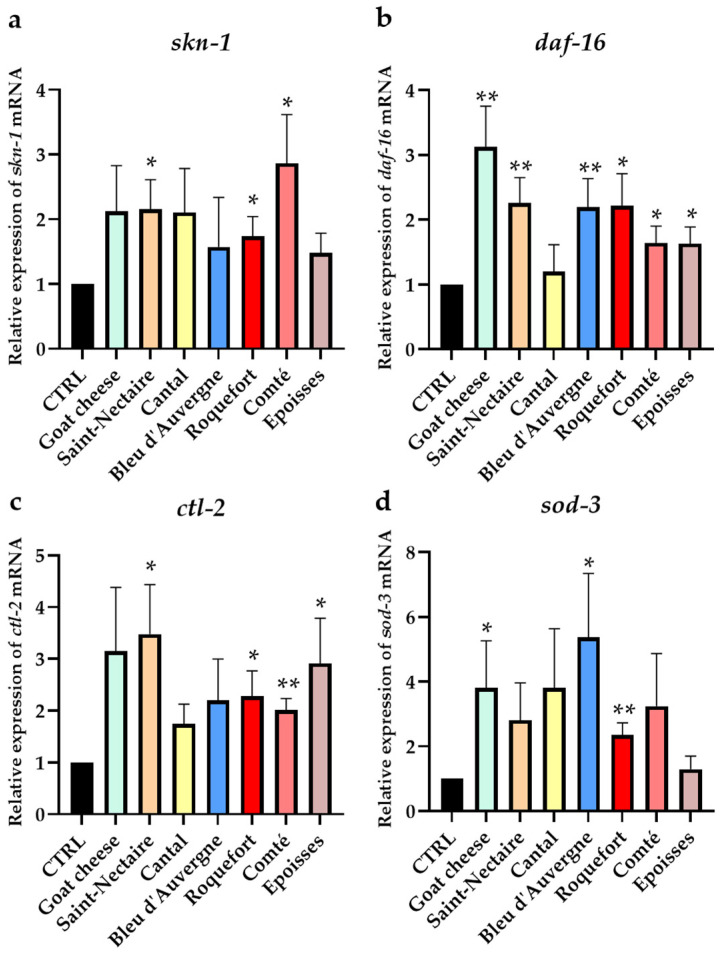
Effect of freeze-dried cheese (FDC) on antioxidant gene expression of *skn-1* (**a**), *daf-16* (**b**), *ctl-2* (**c**) and *sod-3* (**d**). Worms were incubated for 5 days on FDC-supplemented medium or not (CTRL) before oxidative exposure (with H_2_O_2_). Data are expressed as mean ± SEM (*n* = 5). Samples are compared to the relative expression of the control exposed to H_2_O_2_ (CTRL), fixed at 1; * = *p* < 0.05; ** = *p* < 0.01.

To evaluate the impact of cheeses on ROS elimination in vivo, we examined the expression of the two transcription factor-encoding genes *skn-1* and *daf-16*, as well as the expression of the antioxidant enzyme-encoding genes *ctl-2* and *sod-3*. After spending 5 days on media supplemented with FDC or without it (CTRL), the nematodes were stimulated with H_2_O_2_. The gene expression of each condition was compared to the gene expression of the control (CTRL), previously set at the value of 1 (Figure 5).

Interestingly, *skn-1* expression (Figure 5a) was significantly upregulated by 1.74 (*p* < 0.05), 2.15 (*p* < 0.05), and 2.87 (*p* < 0.05) times compared with the control for worms grown in media supplemented with Roquefort, Saint-Nectaire, and Comté, respectively. Concerning the *daf-16* gene (Figure 5b)*,* all cheeses, except Cantal, significantly enhanced its expression from 1.63 for Epoisses (*p* < 0.05) to 3.13 for Goat cheese (*p* < 0.01) compared to the control.

Supplementation with Comté and Roquefort upregulated the expression of the *ctl-2* gene about two times (Figure 5c), by 2.01 (*p* < 0.01) and 2.28 (*p* < 0.05) times, respectively, and Epoisses and Saint-Nectaire about three times, by 2.91 (*p* < 0.05) and 3.48 times (*p* < 0.05), respectively, compared to the control. Worms previously supplemented with freeze-dried Roquefort, Goat cheese, and Bleu d’Auvergne upregulated the expression of the *sod-3* gene by 2.36 (*p* < 0.01), 3.8 (*p* < 0.05), and 5.37 (*p* < 0.05) times the control, respectively (Figure 5d).

## 4. Discussion

In this study, we aimed to evaluate the biological properties of the oxidative process of eight French raw-milk cheeses with varied cheese-making processes, milk types, and ripening (Table 1) to maximize the diversity of their composition and/or microbiota [21,22,23,24,25,26] and potentially their bioactive peptides [16,17,18,19,20]. We used both in vivo and in vitro biological models. We selected the in vivo model *Caenorhabditis elegans*, which shares approximately 60–80% of genes and 12 signaling pathways with humans [46,47]. This organism is commonly used for investigating probiotic effects and screening various molecules or extracts of interest [36,48,49,50], but it is also an ideal model for studying the oxidative process. An exogenous source of ROS, such as hydrogen peroxide, induces a state of oxidative stress in the nematode, leading to a sub-dead or dead condition [51].

To investigate the effect of cheeses on the oxidative process, we conducted survival assays in worms supplemented with 1% of cheese fractions (FDC, ApE, W40, or W70) or without supplementation (CTRL) before exposure to an oxidative medium. These results demonstrated that a 1% supplementation of the medium with each cheese fraction (FDC, apolar, and polar extracts) enhanced the survival rate of *C. elegans* by up to 5.33 times the control (W40 of Goat cheese), thereby protecting it from an oxidative environment. The high mortality of the control nematodes in the oxidative environment might be attributed to an accumulation of oxidative damage, including the presence of ROS in the organism. We hypothesized that cheese fractions (FDC, ApE, W40, and W70) could mitigate this accumulation and associated damage, thereby enhancing the survival of the worms. Our results demonstrated that all cheese fractions decreased ROS accumulation in vivo in *C. elegans* by up to 80% (ApE of Brie de Meaux). As this could be the result of either a reduction in ROS production within the cells and/or an increase in the elimination of free radicals through the activation of metabolic response pathways, we investigated whether our cheeses could influence one or both of these processes.

We conducted an in vitro assay in human cells to determine the effect of the three extracts (ApE, W40, and W70) obtained from the FDC on ROS production. In humans, ROS are produced by immune cells such as leukocytes, particularly neutrophils, which play a role in both host defense and inflammation [52]. We conducted an experiment using PMA-stimulated human circulant leukocytes in vitro. PMA, a phorbol ester analog, activates protein kinase C (PKC) and NADPH oxidase (NOX) in neutrophils and enhances their ROS production [53,54], simulating oxidative stress in vitro. The assay was conducted on the apolar (ApE) and polar (W40 and W70) extracts of each cheese. Remarkably, at least one fraction of each raw-milk cheese significantly reduced ROS production, regardless of the concentration tested. Each ApE reduced ROS production by an average of about 30%, with no dose-dependent effect, suggesting that the maximum effect was reached at 25 µg/mL. Out of the 24 fractions (W40, W70, and ApE) tested, 22 were effective in reducing in vitro ROS production by 10% to 54% (W70 of Roquefort).

**Figure 6:**
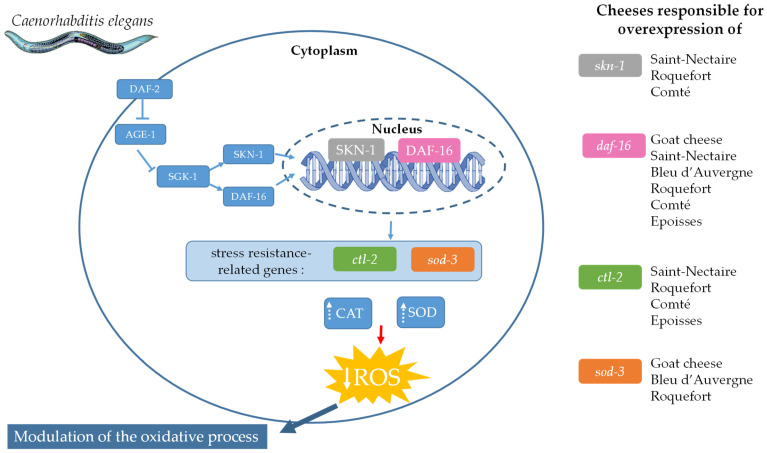
A schematic representation of the mechanisms of action of cheeses in reducing the oxidative process in *Caenorhabditis elegans*. CAT: catalase; SOD: superoxide dismutase; ROS: reactive oxygen species. Inspired by [61].

Another way to protect from ROS accumulation in an oxidative environment is to enhance ROS elimination in cells through the activation of metabolic response pathways. In *C. elegans*, two metabolic pathways are described as having this function: the p38 mitogen-activated protein kinase (p38 MAPK) pathway and the insulin/insulin-like growth factor-1 signaling (IIS) pathways. The p38 MAPK pathway, regulated by the transcription factor SKN-1, the mammalian ortholog of Nrf2, is involved in the immune system and oxidative stress response [55]. When nematodes are stimulated by oxidative stress, SKN-1 is activated and regulates downstream target genes to manage oxidative stress [56]. Another metabolic pathway, the insulin/insulin-like growth factor-1 signaling (IIS) pathway, is described to be involved in immunity, stress resistance, and longevity in *C. elegans* [57,58,59,60]. DAF-16, a FOXO-family transcription factor, is the core gene of this pathway involved in regulating stress resistance. When activated, DAF-16 translocates into the nucleus and is responsible for the regulation of downstream genes implicated in the stress response, such as *ctl-2* and *sod-3*, encoding catalase (CAT) and superoxide dismutase (SOD), respectively, which are implicated in the detoxification of ROS in the organism. Interestingly, although all the cheeses had a beneficial effect on protecting *C. elegans*, they all had different mechanisms to modulate the oxidative process (Figure 6). Among all the cheeses, the FDCs of Saint-Nectaire, Roquefort, and Comté were the only ones that seemed to activate the two molecular signaling pathways: the IIS pathway via *daf-16* overexpression and the p38 MAPK pathway through *skn-1* overexpression. The FDCs of Goat cheese, Roquefort, and Bleu d’Auvergne appeared to only activate the IIS pathway with the overexpression of *daf-16*. Their protective effect seemed to be linked to the activation of SOD, as suggested by the increase in *sod-3* expression. We demonstrated that the protective effect of *C. elegans* against oxidative conditions by the FDCs of Saint-Nectaire, Comté, Roquefort, and Epoisses seemed to involve the activation of the IIS pathway and CAT through the overexpression of *daf-16* and *ctl-2*, respectively. Roquefort seemed to be the only one to provide protection through the overexpression of both molecular pathways (the p38 MAPK and the IIS pathways) but also through the two antioxidant enzymes tested (SOD and CAT) via the overexpression of their genes. Cantal was the only cheese to not overexpress any of the four tested genes (*skn-1*, *daf-16*, *sod-3*, and *ctl-2*), suggesting that its protective effect was provided by other response pathways. Additional research at the protein level could provide further insights into the effects of raw-milk cheeses on the implications of each of these factors.

Since the stress resistance pathways activated in *C. elegans* are conserved in humans, and given that cheeses showed a beneficial effect on oxidative processes in human leukocytes, we can expect a similar effect of raw-milk cheeses on these antioxidant enzyme-encoding genes in human cells. However, further studies are needed to understand the mechanisms of action involved in human cells.

Each raw-milk cheese tested has a beneficial effect on the oxidative process. However, they present differences in antioxidant properties. This observation suggested that there may be variations in the composition of bioactive molecules and their relative concentrations or differences in the effectiveness of molecules between two FDCs. Among the eight cheeses, the Roquefort FDC was the only one able to enhance the expression of all tested genes, suggesting activation of both stress response pathways tested. Coupled with its ability to reduce ROS production in vitro by up to 55% (W70) and ROS accumulation in vivo by up to 60% (FDC), Roquefort appeared to have the best antioxidant properties among all raw-milk cheeses tested. It would be highly insightful to analyze and characterize our cheeses to understand the similarities and differences in their composition and grasp their biological distinctions.

Each raw-milk cheese was fractionated into three extracts: an apolar (ApE) and two polar (W40 and W70) extracts. Interestingly, the three fractions (ApE, W40, and W70) from the same FDC exerted similar protective effects on *C. elegans*. This observation suggests the presence of at least two distinct groups of active molecules, polar and apolar, responsible for protection within each cheese. For all cheeses except Cantal, polar extracts (both W40 and W70) seemed to have higher antioxidant properties than the apolar extract (ApE). These results suggest that polar extracts could contain more molecules responsible for the antioxidant effect or have higher efficiency compared with the apolar extract. Although both polar extracts, W40 and W70, showed different antioxidant properties, W70 extract was more efficient than W40 for seven out of eight tested cheeses. These differences between the two polar extracts suggest that each is composed of a different profile with different efficiencies or concentrations of bioactive molecules. This observation was expected as W70 has been water-extracted at 70 °C, suggesting a more efficient extraction compared with the 40 °C extraction of W40. The chemical characterization of our polar extracts and the comparison of the profiles obtained could help elucidate the differences observed and could lead to a better understanding of the biological activity of these two polar extracts.

Previous studies have suggested that cheese consumption could be involved in the French paradox by reducing systemic inflammation [62,63] and different markers of metabolic syndrome [64,65,66,67] and may improve cardiovascular health. Our study demonstrated that all tested raw-milk cheeses possessed antioxidant properties, not only by reducing ROS production in human leukocytes but also by activating metabolic response pathways in the *C. elegans* model, thus limiting ROS accumulation and its associated damage. These results are consistent with previous in vitro studies on the antioxidant properties of cheeses [21,29,31,68] and go further by showing the antioxidant effects of raw-milk cheeses using both in vitro and in vivo model organisms. These new results highlight the potential of cheese as a key player in the French paradox.

It would be interesting to pursue our study by testing the effects of these raw-milk cheeses on oxidative stress and inflammation-associated diseases or aging, such as cardiovascular diseases [69], neurodegenerative diseases [9], or osteoarticular degenerative diseases [7]. Investigating adipokine signaling might also be of interest, as this pathway is implicated in all these biological processes [70].

## 5. Conclusions

To the best of our knowledge, this study is the first to focus on the biologically comparable effect of French raw-milk cheeses—Goat cheese, Saint-Nectaire, Cantal, Bleu d’Auvergne, Roquefort, Comté, Brie de Meaux, and Epoisses—on oxidative processes using both in vitro and in vivo models. All cheeses studied in this work were able to protect *C. elegans* from oxidative conditions. These results were explained by the biological activity of each raw-milk cheese to reduce the accumulation of ROS in vivo, thus limiting damages associated with both reducing ROS production (in vitro with human leucocytes) and enhancing gene expression of antioxidant enzymes implicated in the elimination of ROS (in vivo with *C. elegans*).

The two types of cheese fractions (apolar and polar) investigated in this study were shown to present similar levels of antioxidative activity, suggesting their respective contribution to the global activity found in whole cheeses. In our study, some mechanistic evidence was shown in the antioxidant gene expression of *C. elegans* and could be pursued in the future by elucidating the chemical composition of raw-milk cheese at the molecular level.

## Figures and Tables

**Table 1:** Characteristics of the different selected French raw-milk cheeses.

Name of the Cheese	Cheese-Making Process	Milk Origin	Paste	Rind	Ripening
Goat cheese	fresh cheese	goat	-	-	3 weeks
Saint-Nectaire	uncooked pressed cheeses	cow	soft cheese	surface molds	6 weeks
Cantal	uncooked cheeses	cow	hard cheese	washed rind	12 to 17 weeks (“entre-deux”)
Bleu d’Auvergne	uncooked unpressed cheeses	cow	internal molds soft cheese	-	8 weeks
Roquefort	uncooked unpressed cheeses	ewe	internal molds soft cheese	-	8 weeks
Comté	cooked cheeses	cow	hard cheese	smear rind	17 weeks
Brie de Meaux	uncooked unpressed cheeses	cow	soft cheese	bloomy rind	5 to 6 weeks (“three-quarters ripened”)
Epoisses	uncooked unpressed cheeses	cow	soft cheese	washed rind	4 weeks

**Table 2:** Targeted *C. elegans* gene primers for qPCR analysis.

Gene Name	Forward Primer (5′-3′)	Reverse Primer (5′-3′)
Y45F10D.4	CGAGAACCCGCGAAATGTCGGA	CGGTTGCCAGGGAAGATGAGGC
*skn-1*	GTTCAATCAACAACAGGTGGATCA	TGGATGTTGGGAACACTCTGTC
*daf-16*	TTCAATGCAAGGAGCATTTG	AGCTGGAGAAACACGAGACG
*ctl-2*	TCCCAGATGGGTACCGTCAT	TCACTCCTTGAGTTGGCTTGA
*sod-3*	CAATTGCTCTCCAACCAGCG	ACCGAAGTCGCGCTTAATAG

## Data Availability

The datasets presented during the current study are available on request from the corresponding author.

## Supplementary Materials

The following supporting information can be downloaded at: https://www.mdpi.com/article/10.3390/nu16121862/s1, Figure S1: Representative GFP fluorescence microscopy images of ROS accumulation in vivo in *C. elegans* from different fractions of Goat cheese; Figure S2: Representative GFP fluorescence microscopy images of ROS accumulation in vivo in *C. elegans* from different fractions of Saint-Nectaire; Figure S3: Representative GFP fluorescence microscopy images of ROS accumulation in vivo in *C. elegans* from different fractions of Cantal; Figure S4: Representative GFP fluorescence microscopy images of ROS accumulation in vivo in *C. elegans* from different fractions of Bleu d’Auvergne; Figure S5: Representative GFP fluorescence microscopy images of ROS accumulation in vivo in *C. elegans* from different fractions of Roquefort; Figure S6: Representative GFP fluorescence microscopy images of ROS accumulation in vivo in *C. elegans* from different fractions of Comté; Figure S7: Representative GFP fluorescence microscopy images of ROS accumulation in vivo in *C. elegans* from different fractions of Brie de Meaux; Figure S8: Representative GFP fluorescence microscopy images of ROS accumulation in vivo in *C. elegans* from different fractions of Epoisses.
