# Supplementary material for: Exploring the Impact of French Raw-Milk Cheeses on Oxidative Process Using Caenorhabditis elegans and Human Leukocyte Models
Source: Nutrients. 2024 Jun 13;16(12):1862. doi: 10.3390/nu16121862 (PMC11206881; doi:10.3390/nu16121862)
Supplement: Supplementary file 1 [file nutrients-16-01862-s001.zip › nutrients-3038246-supplementary.pdf]

nutrients

Supplementary Materials

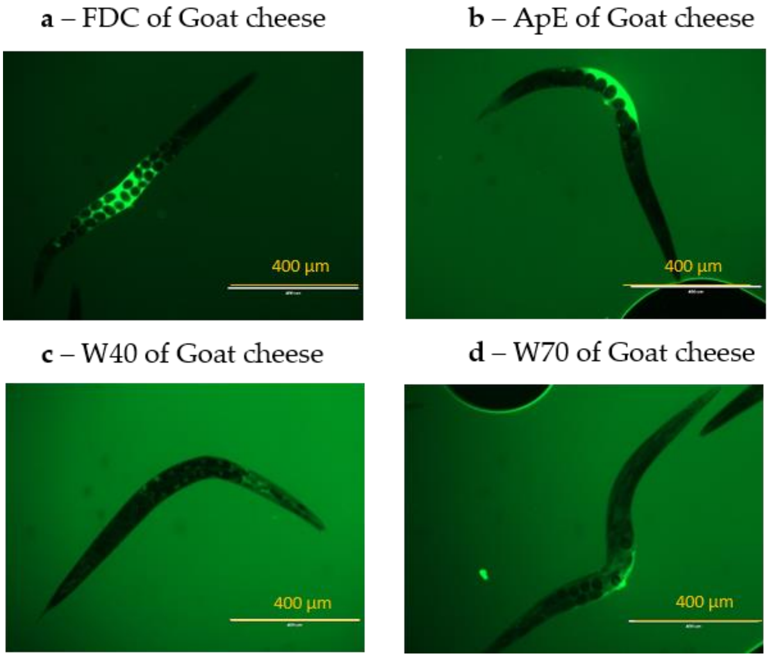

Figure S1. Representative GFP fluorescence microscopy images of ROS accumulation *in vivo* in *C. elegans* from different fractions of Goat cheese. Wild-type N2 strain was cultivated for five days on a standard culture medium supplemented with either FDC **(a)**, ApE (**b**), W40 **(c)** or W70 **(d)** of Goat cheese. After that, worms were placed under oxidative conditions and treated with H_2DCF-DA to assess intracellular ROS levels *in vivo*. Images were acquired at 100x magnification.

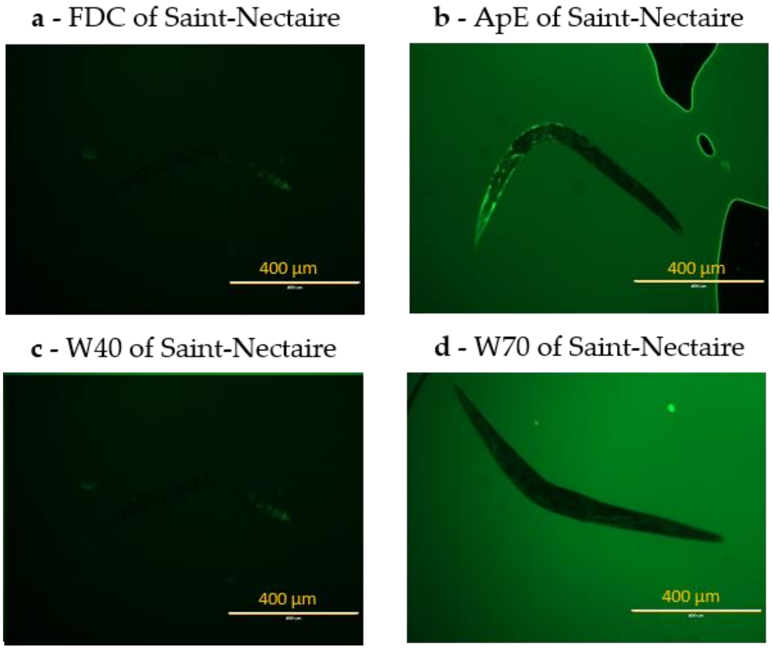

Figure S2. Representative GFP fluorescence microscopy images of ROS accumulation *in vivo* in *C. elegans* from different fractions of Saint-Nectaire. Wild-type N2 strain was cultivated for five days on a standard culture medium supplemented with either FDC **(a)**, ApE (**b**), W40 **(c)** or W70 **(d)** of

Saint-Nectaire. After that, worms were placed under oxidative conditions and treated with H_2DCF-DA to assess intracellular ROS levels *in vivo*. Images were acquired at 100x magnification.

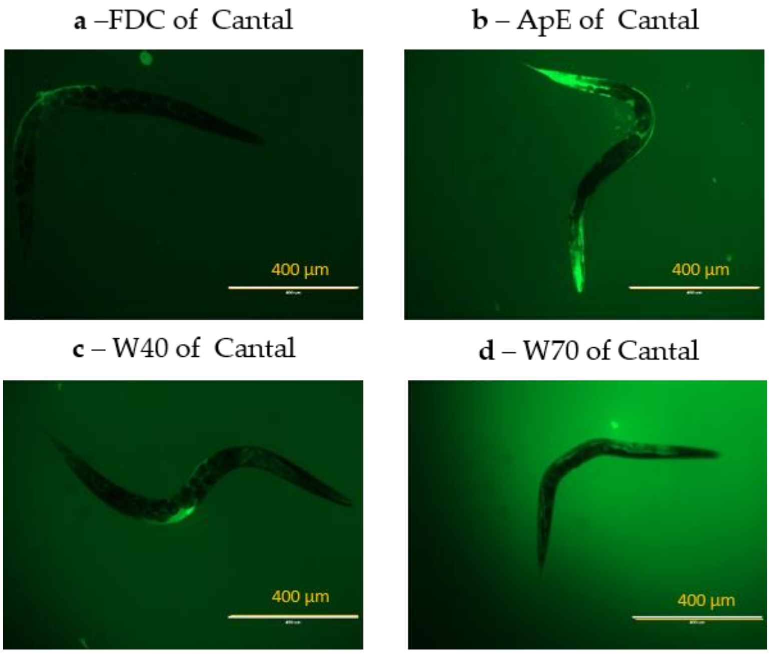

Figure S3. Representative GFP fluorescence microscopy images of ROS accumulation *in vivo* in *C. elegans* from different fractions of Cantal. Wild-type N2 strain was cultivated for five days on a standard culture medium supplemented with either FDC **(a),** ApE (**b**), W40 **(c)** or W70 **(d)** of Cantal. After that, worms were placed under oxidative conditions and treated with H_2DCF-DA to assess intracellular ROS levels *in vivo*. Images were acquired at 100x magnification.

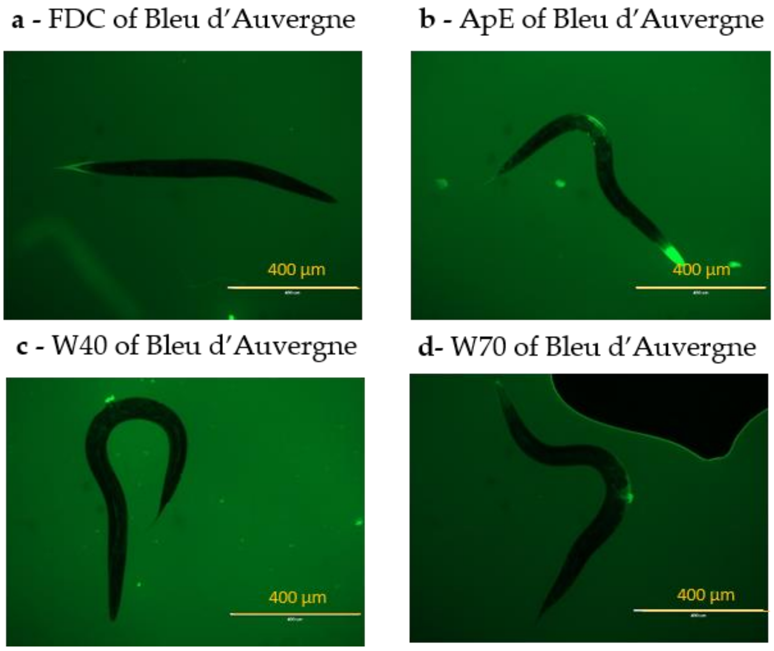

Figure S4. Representative GFP fluorescence microscopy images of ROS accumulation *in vivo* in *C. elegans* from different fractions of Bleu d'Auvergne. Wild-type N2 strain was cultivated for five days on a standard culture medium supplemented with either FDC **(a),** ApE (**b**), W40 **(c)** or W70 **(d)** of Bleu d'Auvergne. After that, worms were placed under oxidative conditions and treated with H_2DCF-DA to assess intracellular ROS levels *in vivo*. Images were acquired at 100x magnification.

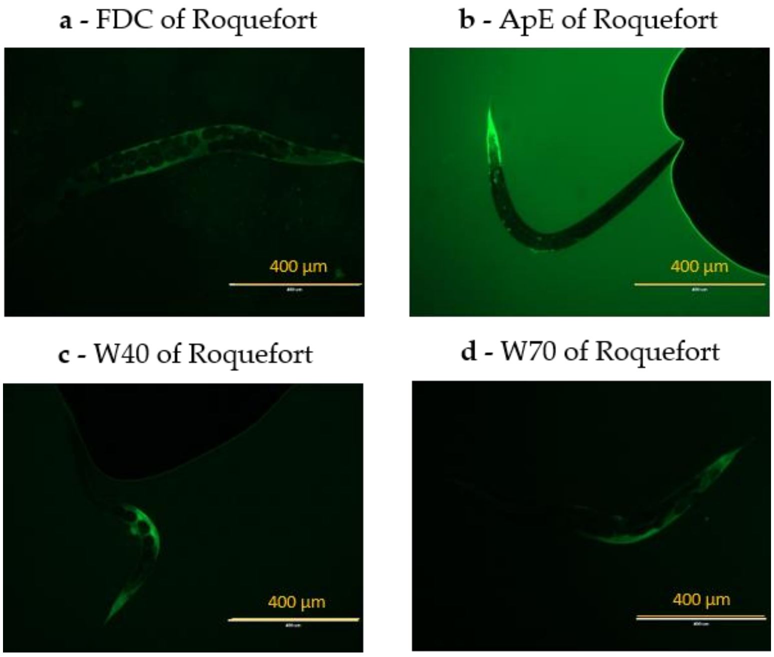

Figure S5. Representative GFP fluorescence microscopy images of ROS accumulation *in vivo* in *C. elegans* from different fractions of Roquefort. Wild-type N2 strain was cultivated for five days on a standard culture medium supplemented with either FDC **(a),** ApE (**b**), W40 **(c)** or W70 **(d)** of Roquefort. After that, worms were placed under oxidative conditions and treated with H_2DCF-DA to assess intracellular ROS levels *in vivo*. Images were acquired at 100x magnification.

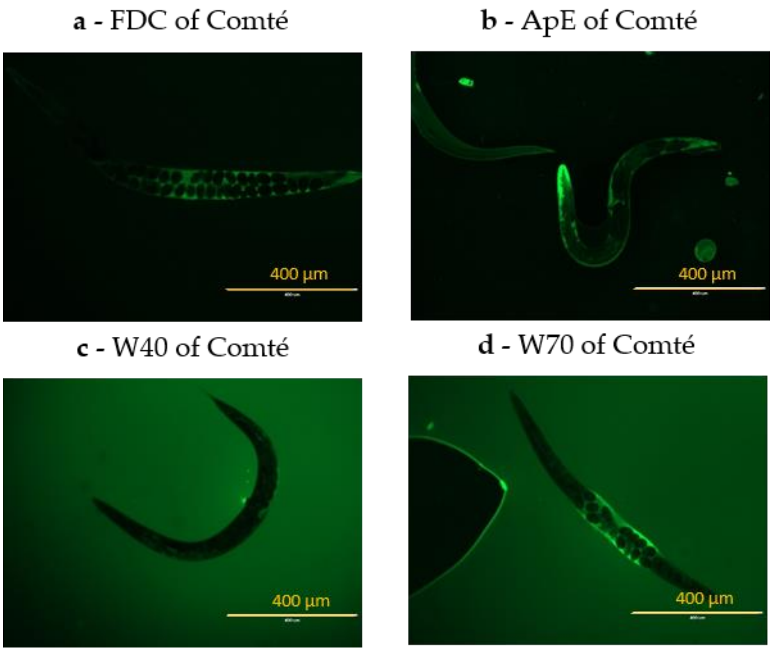

Figure S6. Representative GFP fluorescence microscopy images of ROS accumulation *in vivo* in *C. elegans* from different fractions of Comté. Wild-type N2 strain was cultivated for five days on a standard culture medium supplemented with either FDC **(a),** ApE (**b**), W40 **(c)** or W70 **(d)** of Comté. After that, worms were placed under oxidative conditions and treated with H_2DCF-DA to assess intracellular ROS levels *in vivo*. Images were acquired at 100x magnification.

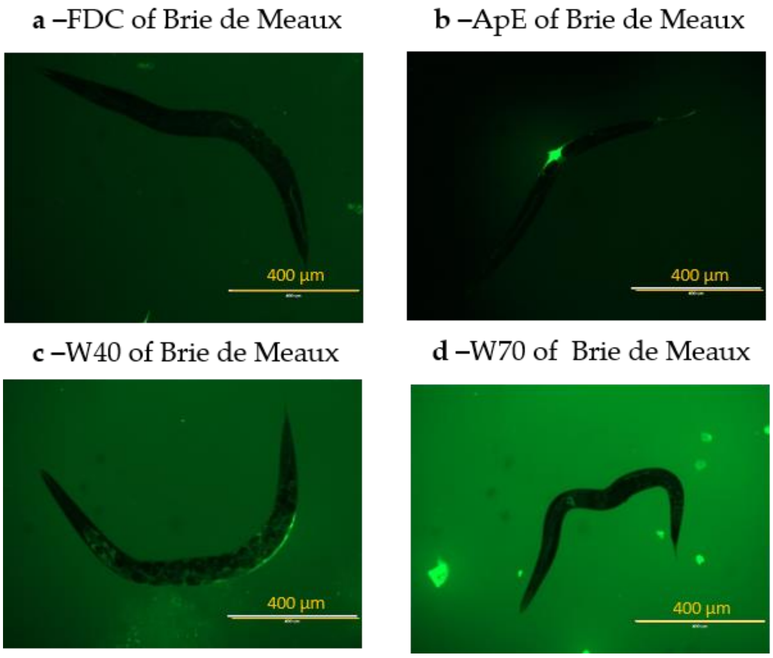

Figure S7. Representative GFP fluorescence microscopy images of ROS accumulation *in vivo* in *C. elegans* from different fractions of Brie de Meaux. Wild-type N2 strain was cultivated for five days on a standard culture medium supplemented with either FDC **(a),** ApE (**b**), W40 **(c)** or W70 **(d)** of Brie de Meaux. After that, worms were placed under oxidative conditions and treated with H2DCF-DA to assess intracellular ROS levels *in vivo*. Images were acquired at 100x magnification.

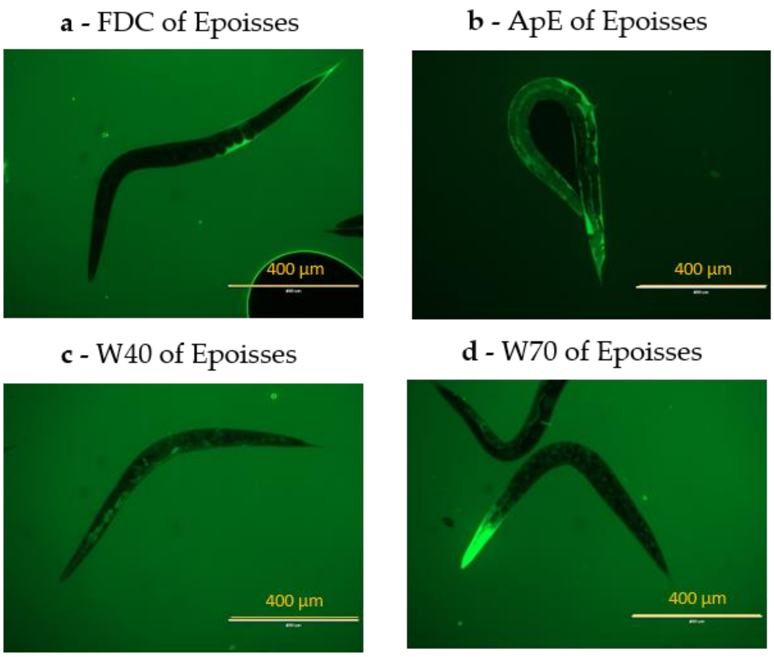

Figure S8. Representative GFP fluorescence microscopy images of ROS accumulation *in vivo* in *C. elegans* from different fractions of Epoisses. Wild-type N2 strain was cultivated for five days on a standard culture medium supplemented with either FDC **(a),** ApE (**b**), W40 **(c)** or W70 **(d)** of Epoisses. After that, worms were placed under oxidative conditions and treated with H2DCF-DA to assess intracellular ROS levels *in vivo*. Images were acquired at 100x magnification.
